# Supplementary material for: Enantioselective OTUD7B fragment discovery through chemoproteomics screening and high-throughput optimisation
Source: Commun Chem. 2025 Jan 15;8:12. doi: 10.1038/s42004-025-01410-8 (PMC11732987; doi:10.1038/s42004-025-01410-8)
Supplement: Supplementary file 2 — Supplementary Information [file 42004_2025_1410_MOESM2_ESM.pdf]

Enantioselective OTUD7B fragment discovery through chemoproteomics screening and high-throughput optimisation

Aini Vuorinen[1,2]†, Cassandra R. Kennedy[2]†, Katherine A. McPhie[2]†, William McCarthy[2], Jonathan Pettinger[3], J. Mark Skehel[1], David House[3], Jacob T. Bush[3]*, Katrin Rittinger[2]*

[1] Proteomics Science Technology Platform, The Francis Crick Institute, London, NW1 1AT, UK.
[2] Molecular Structure of Cell Signalling Laboratory, The Francis Crick Institute, London, NW1 1AT, UK.
[3] Crick-GSK Biomedical LinkLabs, GSK, Stevenage, Hertfordshire, SG1 2NY, UK.

† Authors contributed equally
* Corresponding author

Contents:

SF1 Library screening supplementary data.
SF2 Chemoproteomics supplementary data for all fragments and identified DUBs.
SF3 Chemoproteomics supplementary data for hit fragments against each DUB subfamily.
SF4 Chemoproteomics supplementary volcano plot data for hit fragments **1** – **7.**
SF5 In vitro validation supplementary data.
SF6 Representative examples of intact protein LC-MS spectra for in vitro validation.
SF7 High-throughput chemistry direct-to-biology (HTC-D2B) supplementary data.
SF8 Compounds selected from HTC-D2B for resynthesis and purification for chemoproteomics validation.
SF9 Round 2 chemoproteomics supplementary data.
SF10 Chemoproteomics supplementary data for enantiomers.
SF11 Biochemical and kinetic characterisation of OTUD7A.
SF12 OTUD7B molecular docking and modelling interaction maps.

Supplementary Figure 1 Fragment screening supplementary data. A) Summary of molecular property distributions for the 227 chloroacetamide fragment library: molecular weight, calculated logP (cLogP), fraction of sp3-hybridised centres, chloroacetamide amide motif (‘other’ (blue bar) represents one of each motif: furan, benzothiazole, pthalimide, quinoline, isoxazole, oxadiazole, pyrrole, thiadiazole), and numbers of hydrogen bond donors (HBD), acceptors (HBA), and aromatic rings present per fragment; B) Number of DUB hits for each fragment; C) Number of fragment hits for each DUB. Hit fragments were identified for 32 DUBs across the four different subfamilies (UCH (green), OTU (dark blue), USP (sky blue) and MJD (blue)). The fragment screening was performed with technical replicates (*n* = 3 for compound treated samples, *n* = 44 for DMSO samples).

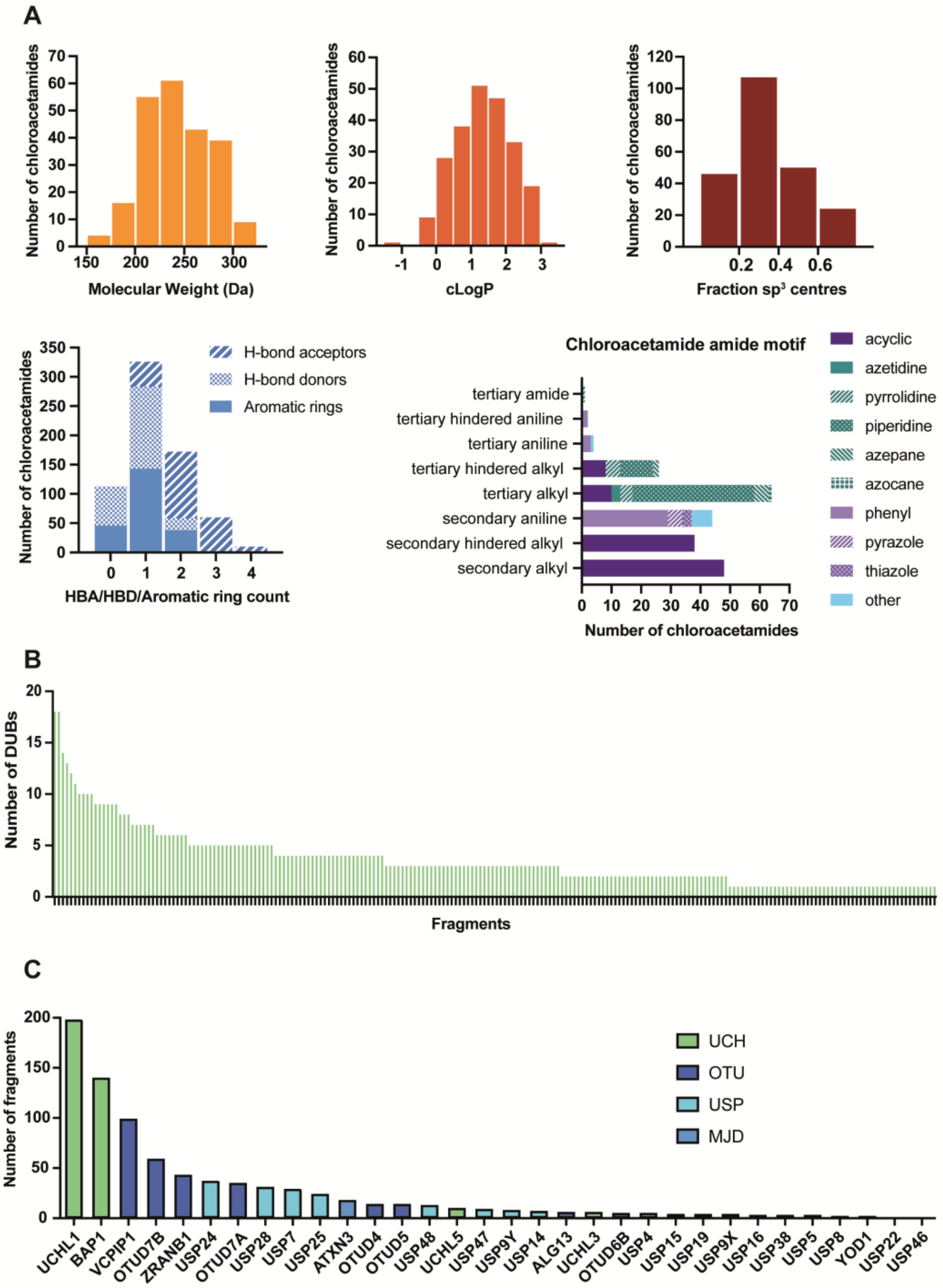

Supplementary Figure 2 Chemoproteomics supplementary data for all fragments and identified DUBs. Heatmap showing chemoproteomics fragment screening data for all 227 fragments against 43 identified DUBs when q-value ≤ 0.05, average $\log_2$ ratio (fragment/DMSO) ≤ 0 and unique peptides ≥ 2. Average $\log_2$ ratios are plotted as absolute values for simplicity. The asterisk (*) denotes a DUB without catalytic cysteine. Hit fragments against each DUB subfamily are presented in Figure 2A and Supplementary Figure 3. The fragment screening was performed with technical replicates (n = 3 for compound treated samples, n = 44 for DMSO samples).

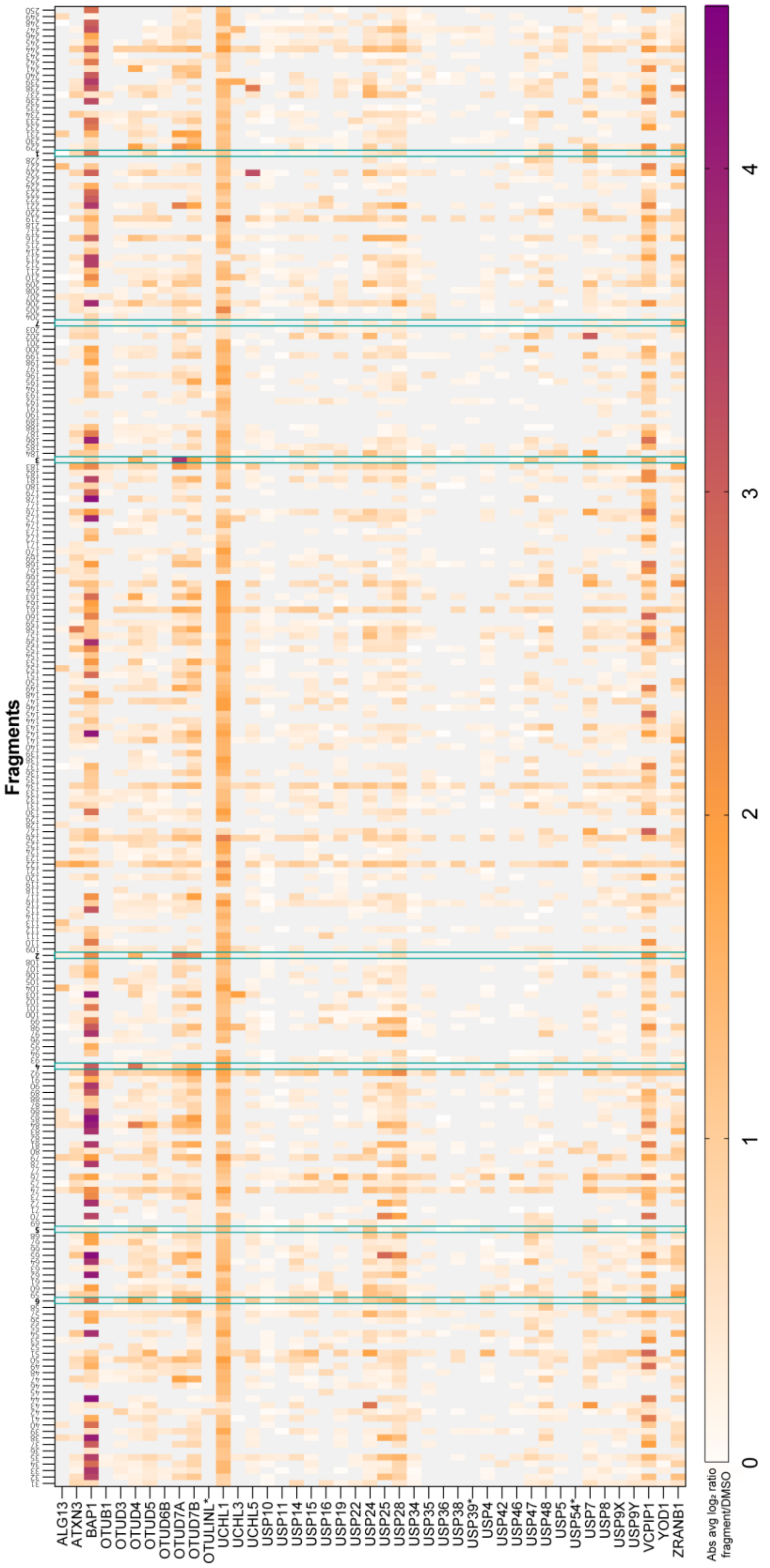
Fragments
ALG13
ATXN3
BAP1
OTUB1
OTUD3
OTUD4
OTUD5
OTUD6B
OTUD7A
OTUD7B
OTULINL*
UCHL1
UCHL3
UCHL5
USP10
USP11
USP14
USP15
USP16
USP19
USP22
USP24
USP25
USP28
USP34
USP35
USP36
USP38
USP39*
USP4
USP42
USP46
USP47
USP48
USP5
USP54*
USP7
USP8
USP9X
USP9Y
VCPIP1
YOD1
ZRANB1
Abs avg log₂ ratio fragment/DMSO
0
1
2
3
4

Supplementary Figure 3 Chemoproteomics fragment screening supplementary data. Identified hit fragments against members of each DUB subfamily showing competed DUBs (bold), quantified DUBs (italics) and DUBs not identified (grey) for A) UCH (209 identified fragment hits), B) USP (67 identified fragment hits), and C) MJD (15 identified fragment hits) DUB subfamilies. Average $\log_2$ ratios are plotted as absolute values for simplicity. Full-length DUB sequences containing catalytic site cysteines were aligned using COBALT[1]. The fragment screening was performed with technical replicates (n = 3 for compound treated samples, n = 44 for DMSO samples).

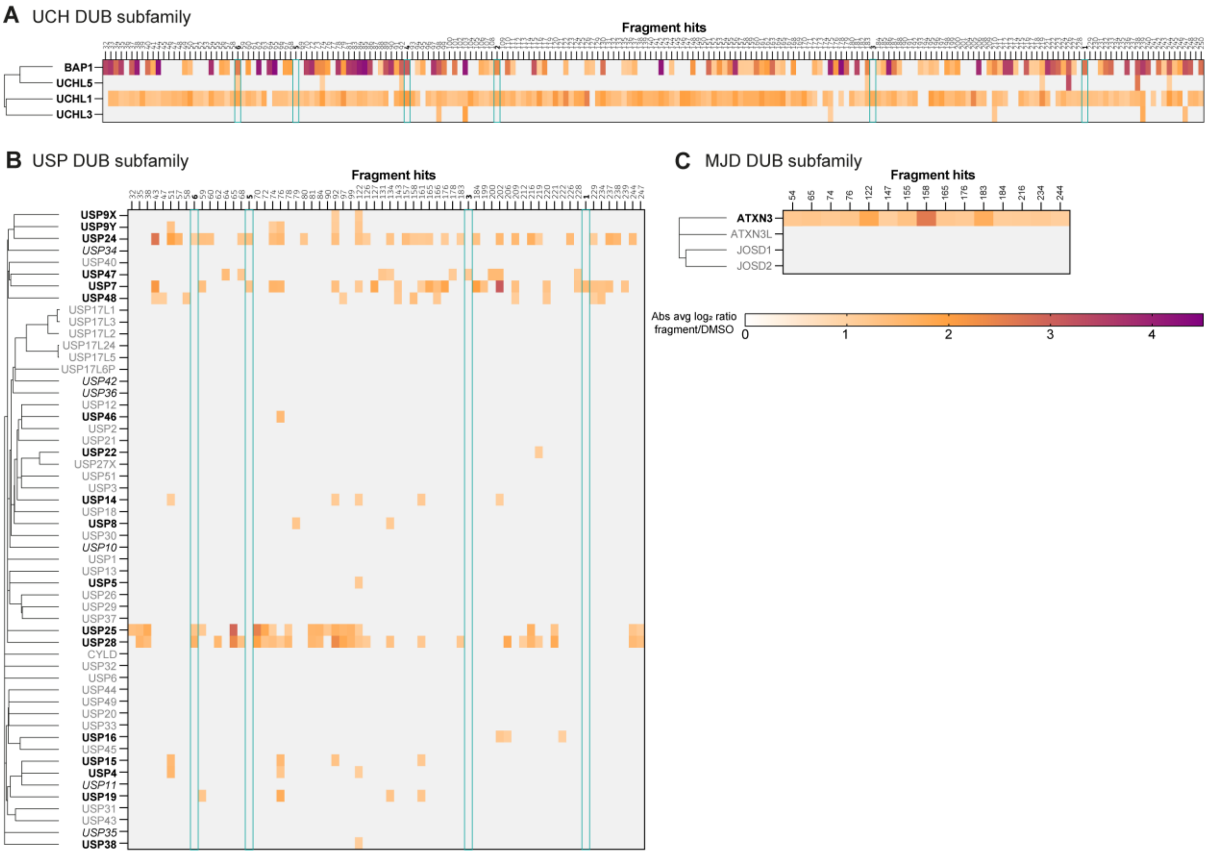

Supplementary Figure 4 Chemoproteomics supplementary data for hit fragments 1 – 7. A) Volcano plots showing chemoproteomics fragment screening data for hit fragments **1** – **7**. Quantified DUBs are highlighted in teal and other proteins are shown in grey. Significantly competed DUBs are labelled. The fragment screening was performed with technical replicates (*n* = 3 for fragment treated samples, *n* = 44 for DMSO samples); B) Table of hit criteria for progression of fragments to in vitro validation and optimisation. For criteria (2), non-OTU family off-target DUBs were considered as hits if average $\log_2$ ratio < -1.0. *For fragment **6**, if average $\log_2$ ratio cutoff is adjusted to < -1.5, 3 non-OTU family off-target DUBs are competed.

A

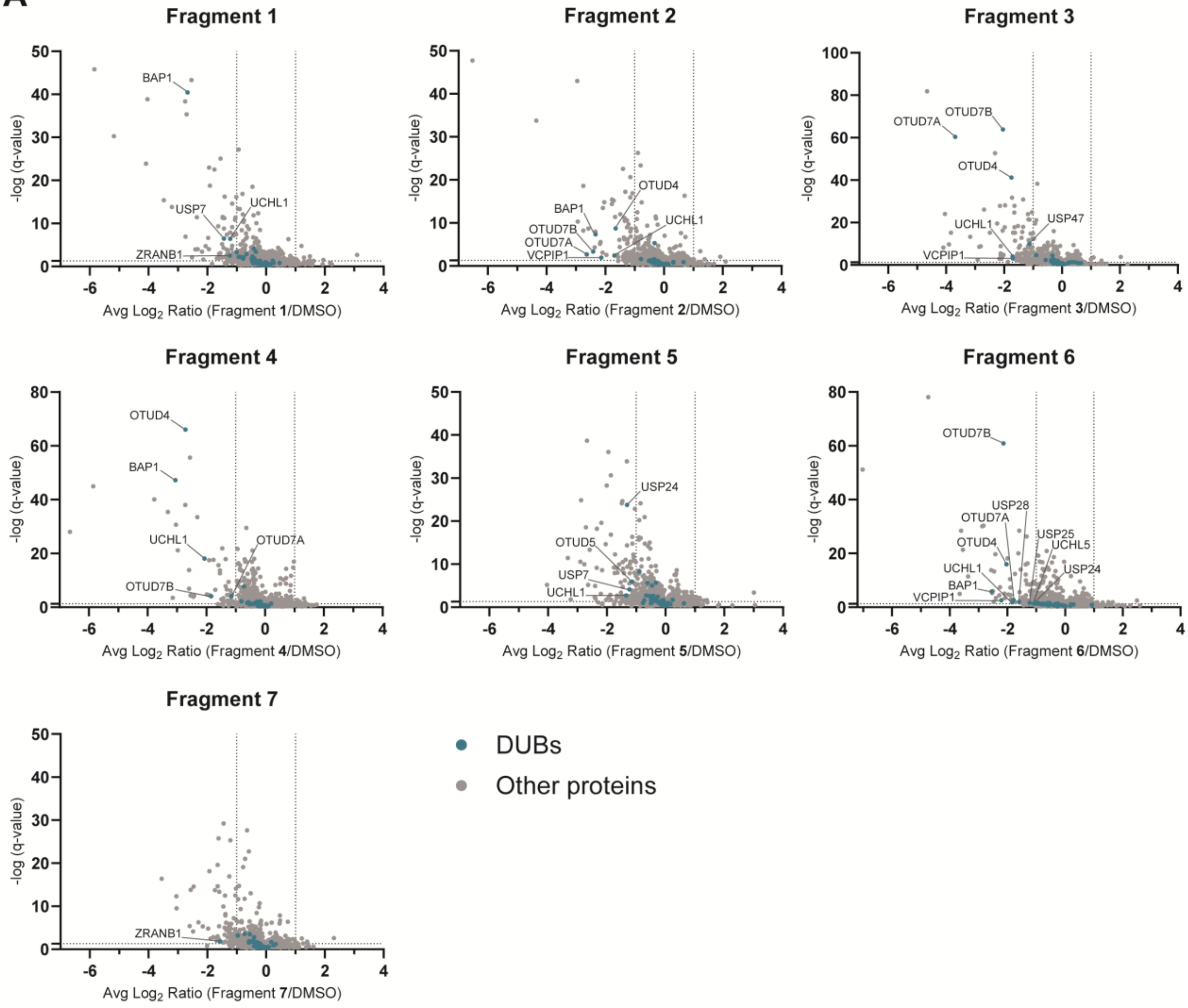

B

		Criteria for selection for *in vitro* validation and optimisation		
**Fragment**	**Original target OTU DUB**	**1) Average log$_2$ ratio (fragment/DMSO) < -1.5**	**2) < 5 off-target DUBs (non-OTU family)**	**3) Chemical structure amenable to HTC**
**1**	ZRANB1	-1.23	3	Yes
**2**	OTUD7A	-2.63	2	Yes
**3**	OTUD7A	-3.69	2	Yes
**4**	OTUD4	-2.71	2	Yes
**5**	OTUD5	-1.15	3	Yes
**6**	OTUD7B	-2.14	6*	Yes
**7**	ZRANB1	-1.57	0	Yes

Supplementary Figure 5 In vitro validation supplementary data. A) Labelling of each DUB by fragments **1** – **7**; B) labelling by each fragment **1** – **7** of each DUB; C) comparison of OTUD5 and activated OTUD5 labelling by each fragment at 200 μM.

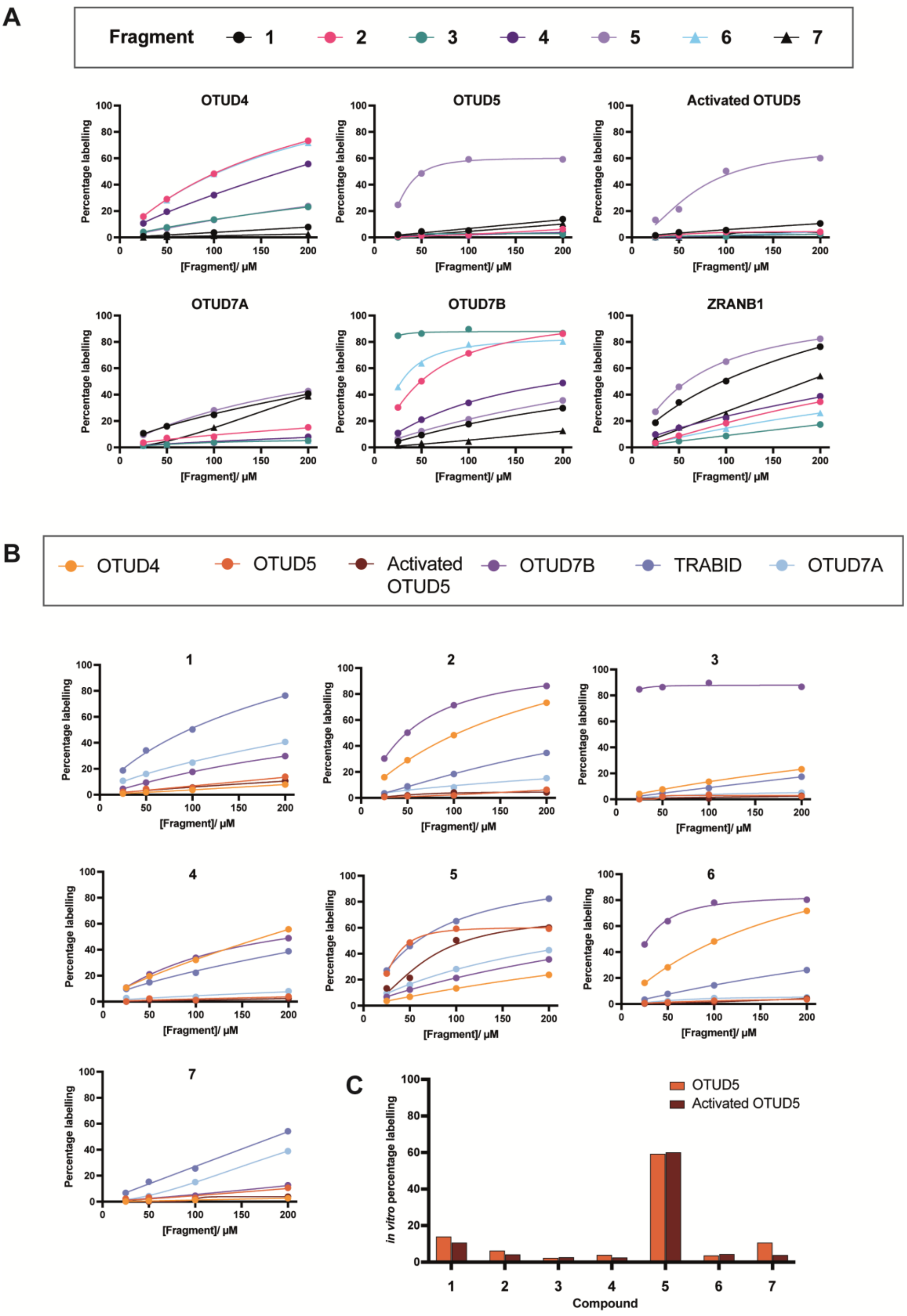

Supplementary Figure 6 Representative examples of intact protein LC-MS spectra for in vitro validation. Deconvoluted intact protein LC-MS spectra for in vitro validation of fragment **6** at 200 µM against each recombinant DUB.

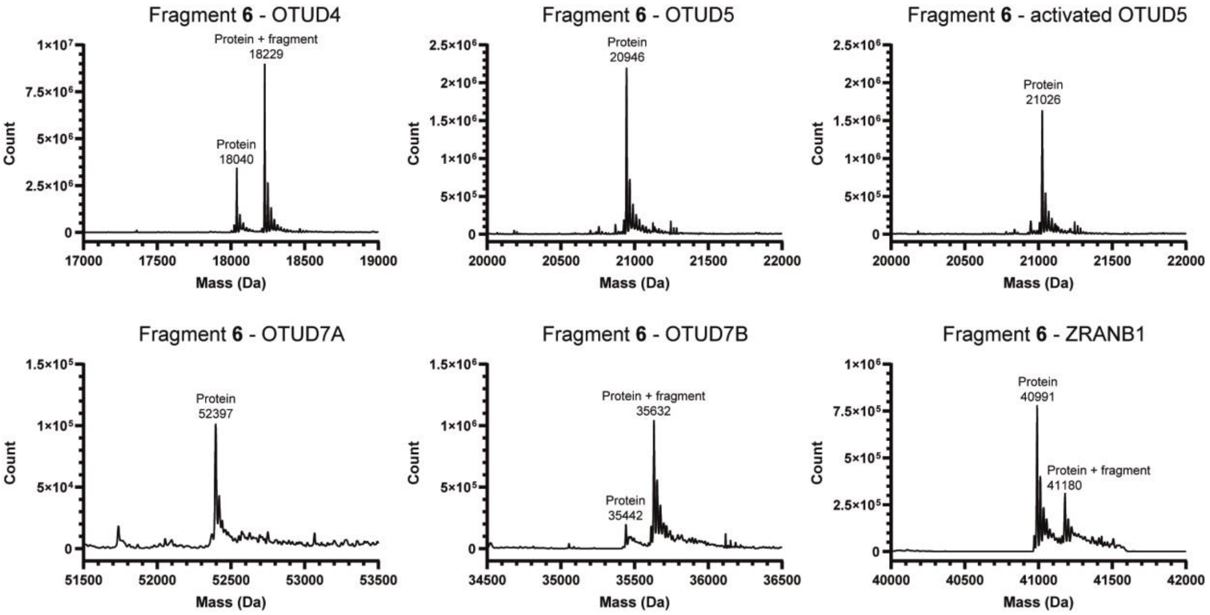

Supplementary Figure 7 High-throughput chemistry direct-to-biology (HTC-D2B) supplementary data. A) Pie-chart of HTC conversion across the plate of 351 chloroacetamides, analysed by LC-MS. Percentage product formation calculated by area under curve (AUC), if correct mass corresponding to desired product is identified; B) Representative examples of raw LC-MS traces for crude HTC reaction mixtures for each category of HTC conversion (> 50% AUC in green, 25-50% AUC in orange, < 25% AUC in red, and 0% AUC (no product mass found) in grey); C) Representative examples of deconvoluted intact protein LC-MS spectra for HTC-D2B protein labelling of HTC compound **28** at 50 μM against each recombinant DUB.

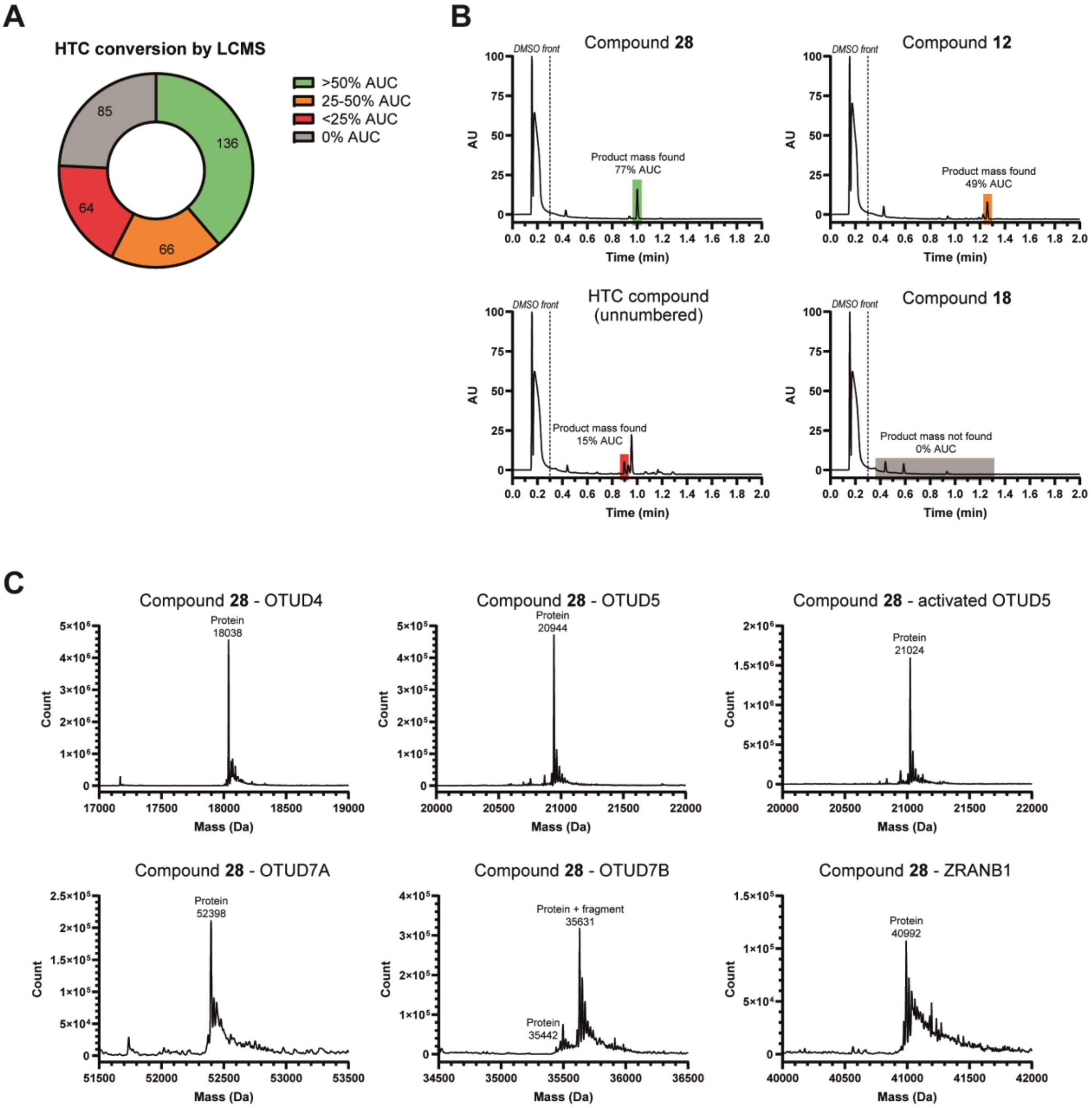

Supplementary Figure 8 Compounds selected from HTC-D2B for resynthesis and purification for chemoproteomics validation. N.B. Compounds **8** – **10** could not be synthesised and purified for follow up.

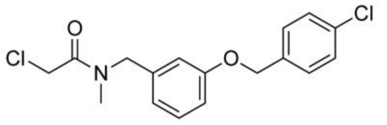

8

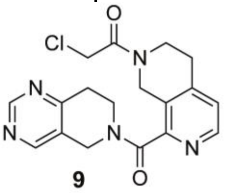

9

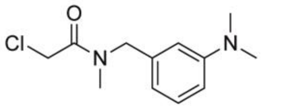

10

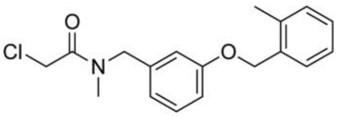

11

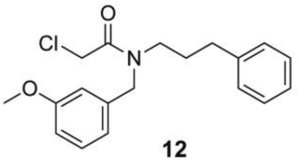

12

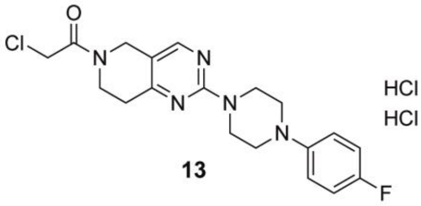

13

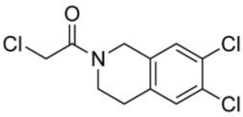

14

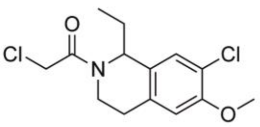

15

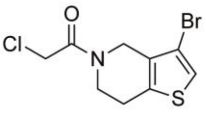

16

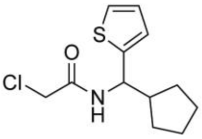

17

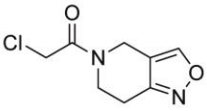

18

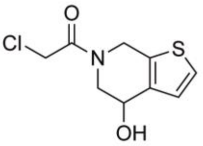

19

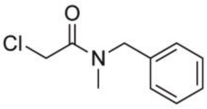

20

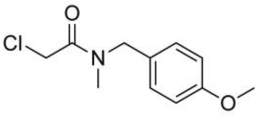

21

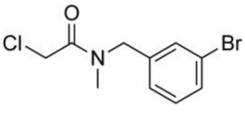

22

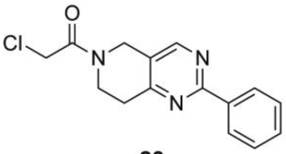

23

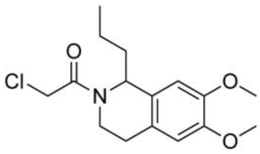

24

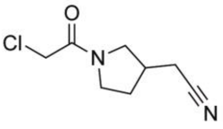

25

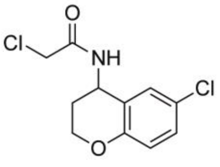

26

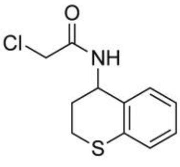

27

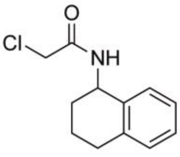

28

Supplementary Figure 9 Round 2 chemoproteomics supplementary data. A) Venn diagram of identified DUBs in round 1 chemoproteomics screen (*n* = 3 for compound treated samples, *n* = 44 for DMSO samples), round 2 chemoproteomics validation (*n* = 3 for compound treated samples, *n* = 26 for DMSO samples) and full concentration-response chemoproteomics validation (*n* = 3 for compound treated samples; *n* = 15 for DMSO samples) experiments; B) Comparison of compound **1** – **7** DUB hits in round 1 and round 2 chemoproteomics; C) Chemoproteomics validation of all fragments at 200 µM (black) and 50 µM (grey) against OTU containing DUBs. OTUD7B can be seen in Figure 4A. The round 2 chemoproteomics validation was performed with technical replicates (*n* = 3 for compound treated samples, *n* = 26 for DMSO samples).

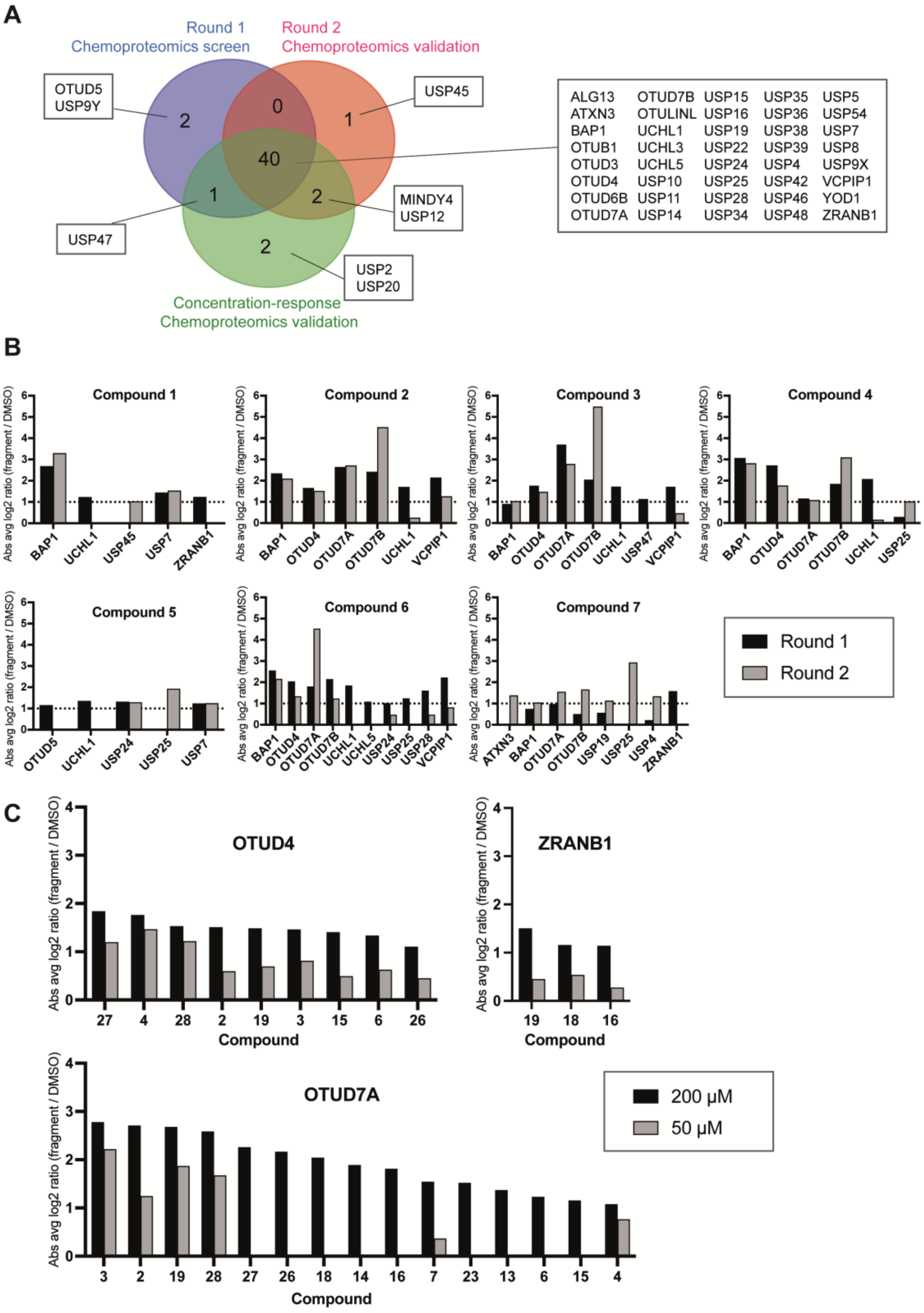

A
Round 1
Chemoproteomics screen
Round 2
Chemoproteomics validation
OTUD5
USP9Y
USP45
USP47
MINDY4
USP12
USP2
USP20
Concentration-response
Chemoproteomics validation
ALG13 ATXN3 BAP1 OTUB1 OTUD3 OTUD4 OTUD6B OTUD7A
OTUD7B OTULINL UCHL1 UCHL3 UCHL5 USP10 USP11 USP14
USP15 USP16 USP19 USP22 USP24 USP25 USP28 USP34
USP35 USP36 USP38 USP39 USP4 USP42 USP46 USP48
USP5 USP54 USP7 USP8 USP9X VCPIP1 YOD1 ZRANB1
B
Compound 1
Compound 2
Compound 3
Compound 4
Compound 5
Compound 6
Compound 7
Abs avg log2 ratio (fragment / DMSO)
Round 1
Round 2
C
OTUD4
ZRANB1
OTUD7A
Compound
Abs avg log2 ratio (fragment / DMSO)
200 µM
50 µM

Supplementary Figure 10 Chemoproteomics supplementary data for enantiomers. Full concentration-response of compounds **28** – **30** by chemoproteomics. A) TE50 values for OTU proteins and hyper-reactive DUBs, quoted to 1 decimal place for values < 100 µM, and to 3 significant figures otherwise. TE50 values for other quantified DUBs could not be determined. 95% CI values are reported to 3 significant figures; B) Concentration-response curves of compounds **28** (grey), **29** (purple) and **30** (teal) for OTUD7A, OTUD4, BAP1, VCPIP1 and USP28. Data are presented as mean ± SEM, *n* = 3 for compound treated samples; *n* = 15 for DMSO samples. Some error bars are too small to be displayed. The curves were fitted with GraphPad Prism 10 using four parameter nonlinear regression with constraints bottom = 0, top = 100; C) Volcano plots showing data at 200 µM for enantiomers **29** and **30** (*n* = 3 for compound treated samples; *n* = 15 for DMSO samples). Quantified DUBs are highlighted in teal and other proteins are shown in grey. Significantly competed DUBs are labelled; D) Heatmap showing IA-DTB chemoproteomics experiment results as competition ratios (CR; DMSO/compound) for compound labelled cysteines. Cysteine labelling by compound was defined based on cut offs (mean $\log_2$ CR ≥ 1 and *p*-value ≤ 0.05) at 200 µM and criteria explained in the methods section. The experiment was performed with technical replicates (*n* = 4 for compound treated samples, *n* = 64 for DMSO samples).

A Proteomics TE50 values (µM)

	**28** (95% CI)	**29** (95% CI)	**30** (95% CI)
OTUD7B	6.9 (5.8 to 8.3)	**3.8** (3.1 to 4.7)	50.5 (39.7 to 64.7)
OTUD7A	N.D.	N.D.	N.D.
OTUD4	36.2 (28.7 to 45.7)	29.6 (24.2 to 36.3)	55.2 (42.4 to 73.6)
BAP1	139 (107 to 198)	138 (101 to 221)	133 (97.7 to 218)
VCPIP1	177 (111 to 397)	579 (179 to 70200)	221 (119 to 632)
USP28	360 (220 to 961)	210 (146 to 397)	N.D.

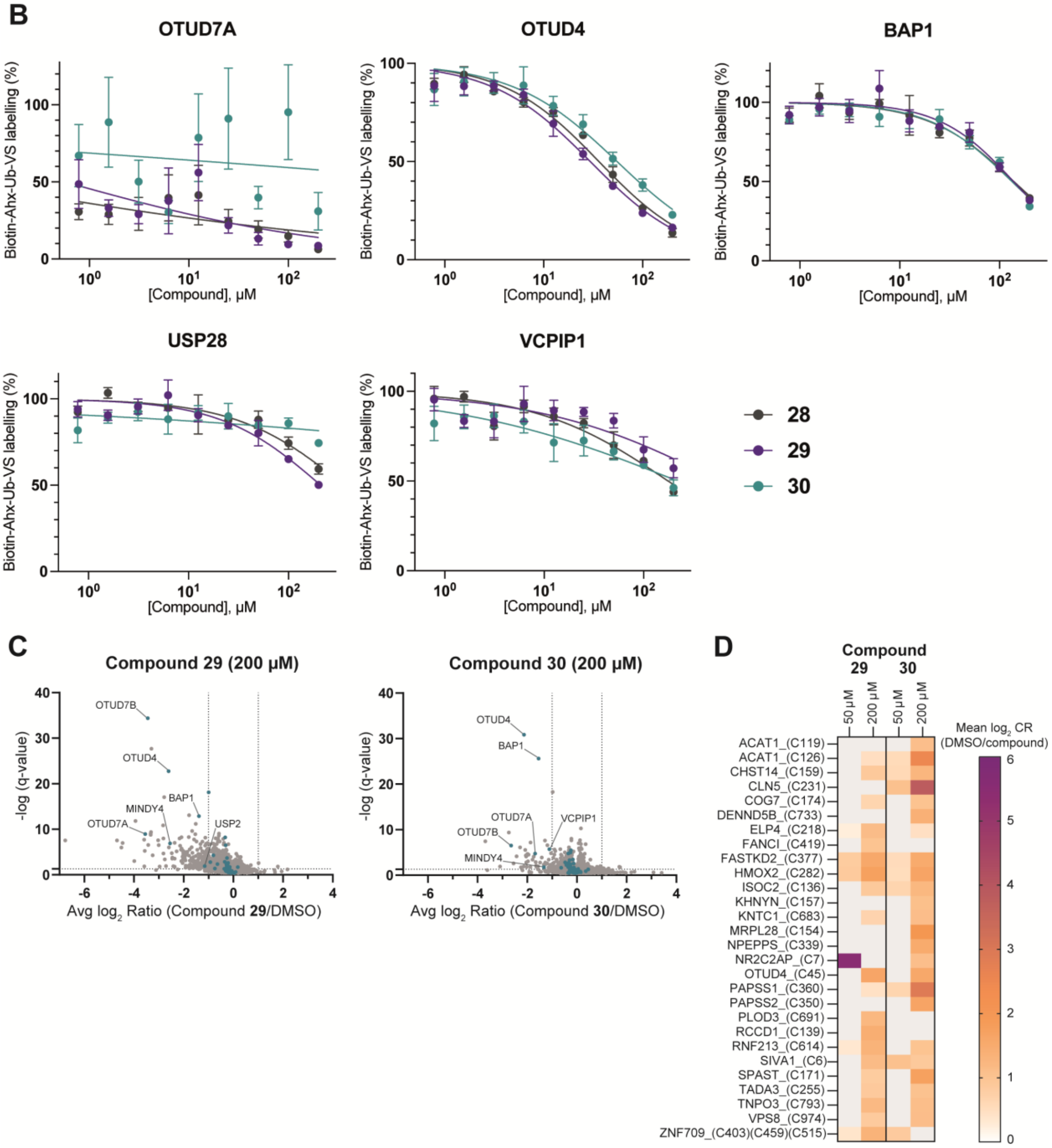

Supplementary Figure 11 Biochemical and kinetic characterisation of OTUD7A. A) Enzymatic inhibition assay data for compounds **28** (grey), **29** (purple) and **30** (teal) against OTUD7A. Data are presented as mean ± SD, *n* = 3. Some error bars are too small to be displayed. The curves were fitted with GraphPad Prism 9 using four parameter nonlinear regression with constraints bottom = 0, top = 100. IC50 values and 95% CI values are quoted to 3 significant figures, or 1 decimal place (for OTUD7B); B) Time courses (0 – 18 hours) of compound labelling (100 – 1.56 μM) against OTUD7A (0.5 μM), performed in technical triplicate (shown on graphs). Labelling percentages were plotted against time in GraphPad Prism 10, and curves were fitted separately for each replicate using straight line fit, no constraints. k_{obs} and k_{inact}/K_I values could not be derived from these straight line plots.

A

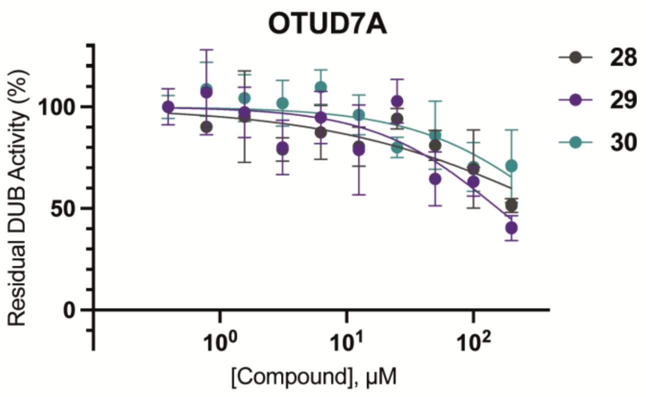

	OTUD7A IC50 (95% CI)	OTUD7B IC50 (95% CI)
**28**	458 μM (163 to 10700)	8.1 μM (7.3 to 8.9)
**29**	157 μM (87.5 to 614)	3.5 μM (3.3 to 3.8)
**30**	443 μM (225 to 2210)	58.3 μM (46.8 to 71.9)

B

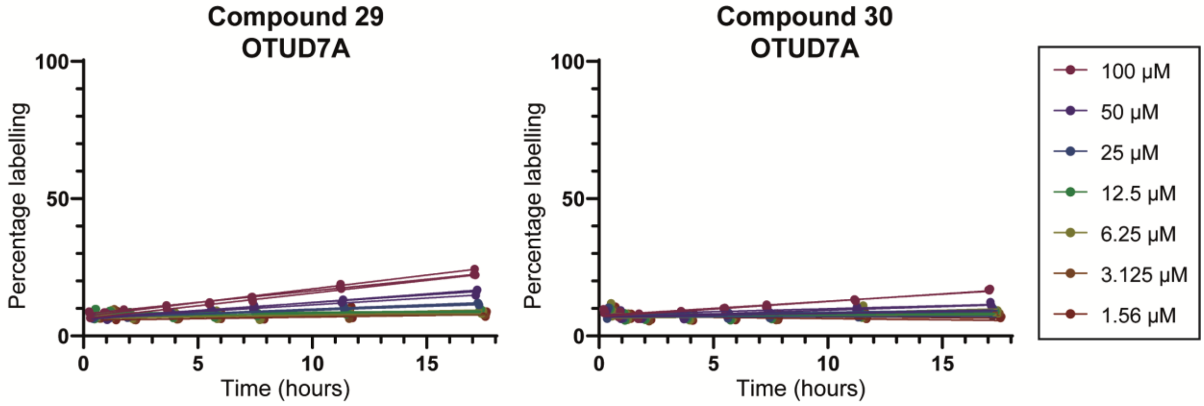

Supplementary Figure 12 OTUD7B molecular docking and modelling interaction maps. Covalent molecular docking of compounds **29** (purple) and **30** (teal) into OTUD7B active site, at Cys194 (using PDB 5LRU[2]). A) Modelled poses for compounds **29** (purple) and; B) **30** (teal) binding to OTUD7B in stick view, and; C) Space-filling model for compound **29** (purple) and; D) **30** (teal); E) 2D-interaction maps for modelled poses for compounds **29** (purple) and; F) **30** (teal) binding to OTUD7B.

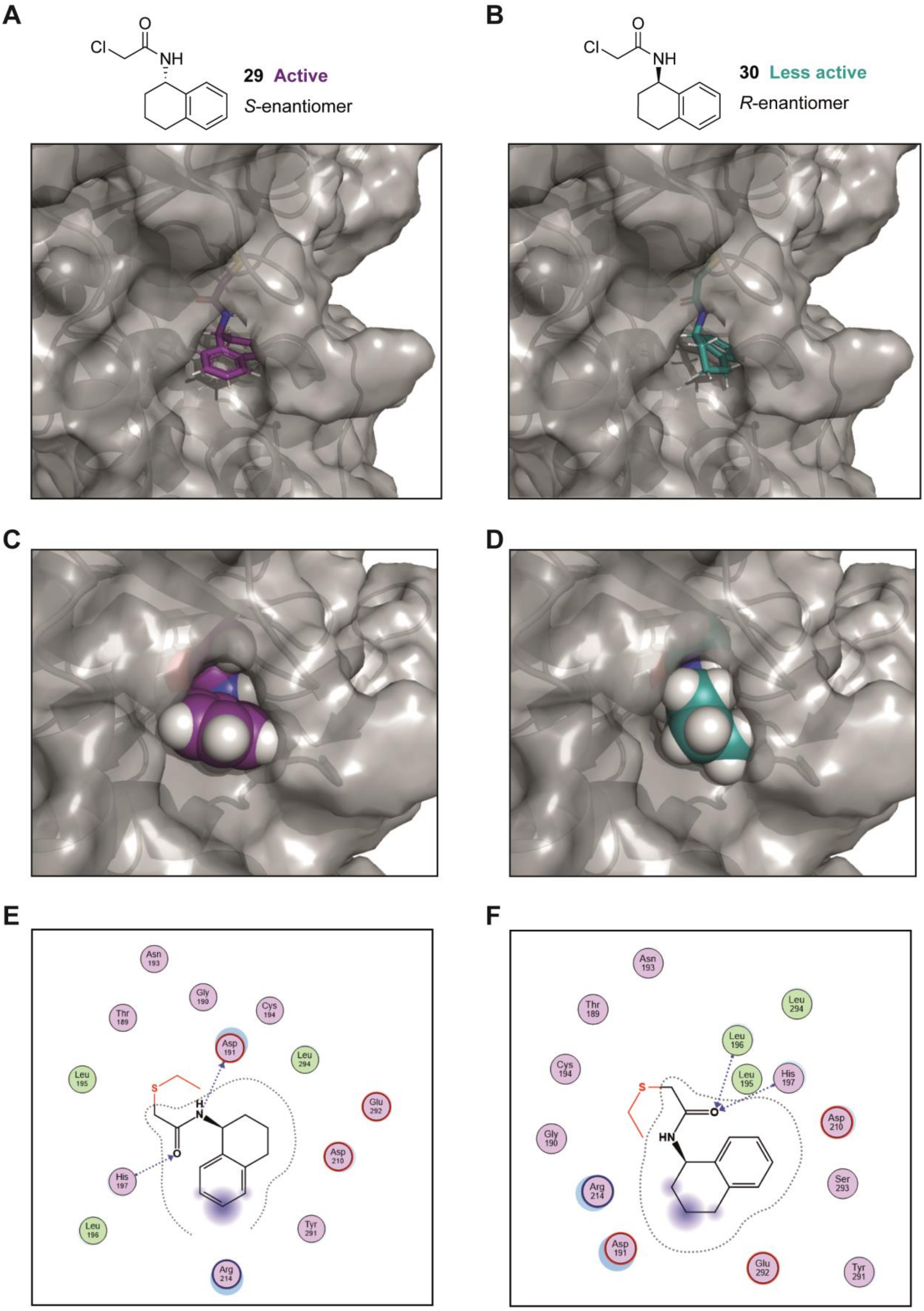
A
Cl
O
NH
29 Active
S-enantiomer
B
Cl
O
NH
30 Less active
R-enantiomer
C
D
E
Asn 193
Thr 189
Gly 190
Cys 194
Asp 191
Leu 294
Leu 195
S
H
N
Glu 292
O
Asp 210
His 197
Leu 196
Tyr 291
Arg 214
F
Asn 193
Thr 189
Leu 294
Leu 196
Cys 194
Leu 195
His 197
S
O
Asp 210
Gly 190
HN
Arg 214
Ser 293
Asp 191
Glu 292
Tyr 291

Supplementary References

1. Papadopoulos, J. S. & Agarwala, R. COBALT: Constraint-based alignment tool for multiple protein sequences. *Bioinformatics* **23**, 1073–1079 (2007).
2. Mevissen, T. E. T. *et al.* Molecular basis of Lys11-polyubiquitin specificity in the deubiquitinase Cezanne. *Nature* **538**, 402–405 (2016).
