# Supplementary material for: Enantioselective OTUD7B fragment discovery through chemoproteomics screening and high-throughput optimisation
Source: Commun Chem. 2025 Jan 15;8:12. doi: 10.1038/s42004-025-01410-8 (PMC11732987; doi:10.1038/s42004-025-01410-8)
Supplement: Supplementary file 3 — Description of Additional Supplementary Files [file 42004_2025_1410_MOESM3_ESM.pdf]

Description of Additional Supplementary Files

File name- Supplementary Data 1

File description- Chemoproteomics data for 227 fragment library screen.

File name- Supplementary Data 2

File description- Chemoproteomics data for round 2 chemoproteomics profiling.

File name- Supplementary Data 3

File description- Chemoproteomics data for concentration-response experiment for compounds 29 and 30.

File name- Supplementary Data 4

File description- SD4 Chemoproteomics data for IA-DTB profiling for compounds 29 and 30.

File name- Supplementary Data 5

File description- List of fragment library compounds, and commercial compounds.

File name- Supplementary Data 6

File description- diaPASEF isolation windows for chemoproteomics data acquisition.

File name- Supplementary Data 7

File description- Biochemical assays raw FI counts.

File name- Supplementary Data 8

File description- Kinetics single labelling percentages.
