# Supplementary material for: Enantioselective OTUD7B fragment discovery through chemoproteomics screening and high-throughput optimisation
Source: Commun Chem. 2025 Jan 15;8:12. doi: 10.1038/s42004-025-01410-8 (PMC11732987; doi:10.1038/s42004-025-01410-8)
Supplement: Supplementary file 12 — Reporting Summary [file 42004_2025_1410_MOESM12_ESM.pdf]

nature portfolio

Corresponding author(s): Katrin Rittinger and Jacob Bush

Last updated by author(s): Nov 29, 2024

Reporting Summary

Nature Portfolio wishes to improve the reproducibility of the work that we publish. This form provides structure for consistency and transparency in reporting. For further information on Nature Portfolio policies, see our Editorial Policies and the Editorial Policy Checklist.

Statistics

For all statistical analyses, confirm that the following items are present in the figure legend, table legend, main text, or Methods section.

n/a	Confirmed	
☐	☒	The exact sample size (*n*) for each experimental group/condition, given as a discrete number and unit of measurement
☐	☒	A statement on whether measurements were taken from distinct samples or whether the same sample was measured repeatedly
☐	☒	The statistical test(s) used AND whether they are one- or two-sided<br>*Only common tests should be described solely by name; describe more complex techniques in the Methods section.*
☐	☒	A description of all covariates tested
☐	☒	A description of any assumptions or corrections, such as tests of normality and adjustment for multiple comparisons
☐	☒	A full description of the statistical parameters including central tendency (e.g. means) or other basic estimates (e.g. regression coefficient) AND variation (e.g. standard deviation) or associated estimates of uncertainty (e.g. confidence intervals)
☐	☒	For null hypothesis testing, the test statistic (e.g. *F*, *t*, *r*) with confidence intervals, effect sizes, degrees of freedom and *P* value noted<br>*Give P values as exact values whenever suitable.*
☒	☐	For Bayesian analysis, information on the choice of priors and Markov chain Monte Carlo settings
☒	☐	For hierarchical and complex designs, identification of the appropriate level for tests and full reporting of outcomes
☒	☐	Estimates of effect sizes (e.g. Cohen's *d*, Pearson's *r*), indicating how they were calculated

Our web collection on statistics for biologists contains articles on many of the points above.

Software and code

Policy information about availability of computer code

Data collection	All mass spectra for chemoproteomics experiments were acquired using an Evosep One LC coupled with to a Bruker timsTOF Pro 2 Mass Spectrometer, with corresponding software provided by Bruker. Site identification mass spectra was acquired using an Ultimate 3000 RSLCnano system coupled with an Orbitrap Fusion Lumos Tribrid mass spectrometer with corresponding software from Thermo Fisher Scientific.
Data analysis	All chemoproteomics data was searched against project specific libraries using Pulsar search engine in Spectronaut (v. 16). Site identification data was searched against the Swissprot database using Mascot software 15 (Matrix Science, Version 2.8.0). Molecular docking was performed using Molecular Operating Environment (MOE, v2020.0901). Figures were generated either using Biorender (https:// www.biorender.com/), PyMOL (v2.5.4), or GraphPad Prism (v9 and v10).

For manuscripts utilizing custom algorithms or software that are central to the research but not yet described in published literature, software must be made available to editors and reviewers. We strongly encourage code deposition in a community repository (e.g. GitHub). See the Nature Portfolio guidelines for submitting code & software for further information.

Data

Policy information about availability of data

All manuscripts must include a data availability statement. This statement should provide the following information, where applicable:

- Accession codes, unique identifiers, or web links for publicly available datasets
- A description of any restrictions on data availability
- For clinical datasets or third party data, please ensure that the statement adheres to our policy

The proteomics data has been submitted to the ProteomeXchange Consortium via the PRIDE database under the identifiers: PXD054883 and PXD057851

Human research participants

Policy information about studies involving human research participants and Sex and Gender in Research.

Reporting on sex and gender	N/A
Population characteristics	N/A
Recruitment	N/A
Ethics oversight	N/A

Note that full information on the approval of the study protocol must also be provided in the manuscript.

Field-specific reporting

Please select the one below that is the best fit for your research. If you are not sure, read the appropriate sections before making your selection.

☒ Life sciences ☐ Behavioural & social sciences ☐ Ecological, evolutionary & environmental sciences

For a reference copy of the document with all sections, see nature.com/documents/nr-reporting-summary-flat.pdf

Life sciences study design

All studies must disclose on these points even when the disclosure is negative.

Sample size	No statistical method was used to determine sample size. For DUB chemoproteomics experiments, all compound-treated conditions were run in technical triplicate and at least 15 untreated (DMSO) samples were run. For IA-DTB chemoproteomics experiment all compound-treated conditions were run in technical quadruplicate and 64 untreated (DMSO) samples were run. Site identification experiment was done as singlicate.
Data exclusions	No exclusion criteria were pre-established. Data quality of each spectra was determined by interpreting quality control criteria for individual runs. For DUB chemoproteomics proteins with less than 2 unique peptides and JAMM subfamily members, which do not contain catalytic cysteine, were removed from further analysis. For peptides that were not associated with a human proteome (according to UniProt) or that matched an entry in the contaminant database were removed from further analysis.
Replication	All data was acquired with technical (n=3 or n=4) replicates, and all observations are made in the context of any variability present in this experiments.
Randomization	Samples were randomised prior to LC-MS data acquisition.
Blinding	Blinding was not necessary as for the experiments described in the manuscript, as scientist bias could not have any impact on the data or the final measurement.

Reporting for specific materials, systems and methods

We require information from authors about some types of materials, experimental systems and methods used in many studies. Here, indicate whether each material, system or method listed is relevant to your study. If you are not sure if a list item applies to your research, read the appropriate section before selecting a response.

Materials & experimental systems

n/a	Involved in the study
☒	☐ Antibodies
☐	☒ Eukaryotic cell lines
☒	☐ Palaeontology and archaeology
☒	☐ Animals and other organisms
☒	☐ Clinical data
☒	☐ Dual use research of concern

Methods

n/a	Involved in the study
☒	☐ ChIP-seq
☒	☐ Flow cytometry
☒	☐ MRI-based neuroimaging

Eukaryotic cell lines

Policy information about cell lines and Sex and Gender in Research

Cell line source(s)	HEK293T (original origin: female human embyro) cells were obtained from the Francis Crick Institute Cell Services STP.
Authentication	Cells were authenticated by STR profiling by Francis Crick Institute Cell Services STP.
Mycoplasma contamination	All cells were negative for mycoplasma contamination, based on fluorescence staining, agar culture, and PCR testing performed by Francis Crick Institute Cell Services STP.
Commonly misidentified lines (See ICLAC register)	No commonly misidentified lines were used.
